# Stochastic inequalities and applications to dynamics analysis of a novel SIVS epidemic model with jumps

**DOI:** 10.1186/s13660-017-1418-8

**Published:** 2017-06-15

**Authors:** Xiaona Leng, Tao Feng, Xinzhu Meng

**Affiliations:** 1 College of Mathematics and Systems Science, Shandong University of Science and Technology, Qingdao, 266590 P.R. China; 2 State Key Laboratory of Mining Disaster Prevention and Control Co-founded by Shandong Province and the Ministry of Science and Technology, Shandong University of Science and Technology, Qingdao, 266590 P.R. China

**Keywords:** stochastic SIVS epidemic model, Lévy jumps, persistence in mean, double epidemic diseases, Doob’s martingale inequality, Hölder’s inequality

## Abstract

This paper proposes a new nonlinear stochastic SIVS epidemic model with double epidemic hypothesis and Lévy jumps. The main purpose of this paper is to investigate the threshold dynamics of the stochastic SIVS epidemic model. By using the technique of a series of stochastic inequalities, we obtain sufficient conditions for the persistence in mean and extinction of the stochastic system and the threshold which governs the extinction and the spread of the epidemic diseases. Finally, this paper describes the results of numerical simulations investigating the dynamical effects of stochastic disturbance. Our results significantly improve and generalize the corresponding results in recent literatures. The developed theoretical methods and stochastic inequalities technique can be used to investigate the high-dimensional nonlinear stochastic differential systems.

## Introduction

Mathematical inequalities are widely used in many fields of mathematical analysis, especially differential systems [1–5]. Recently, the inequality technique was applied to stochastic differential systems [6–11], impulsive differential systems [12–21], and impulsive stochastic differential systems [22], thus some new results have been obtained.

As an important factor threatening the safety of human life and property, the investigation of epidemic has received extensive attention from experts in various fields [23–27]. Generally speaking, medical researchers often use observation and experimental methods to study the behavior of epidemics. Recently, however, a number of experts in the field of mathematics have also been interested in the study of epidemics. They have used mathematical methods to analyze the spread and control of epidemics [28–31]. Kermack and McKendrick’s pioneering work on the development of an epidemic disease is one of the typical examples. They established an SIS compartment model and proposed the famous threshold theory, which has laid a solid foundation for the study of the dynamics of infectious diseases [30].

The SIS model based on the deterministic ordinary differential equation is given by

1 $$ \textstyle\begin{cases} \dot{S}(t)= A-\beta S(t)I(t)-uS(t)+rI(t), \\ \dot{I}(t)= \beta S(t)I(t)-(u+\alpha+r)I(t). \end{cases} $$

In system (1), $\beta S(t)$ represents the number of people infected by a patient within a unit time at *t*. But in reality, the number of people who can be exposed to a patient at a time is limited. To this end, some authors have introduced a saturated infection rate to study the dynamic behavior of the disease [32–34]. In addition, all creatures on the earth are infected by a variety of environmental noises, of course, the disease is no exception. Motivated by this, some scholars have studied the infection system with environmental noises (such as Brownion noise, Markov noise and Lévy noise) [35–38]. Meanwhile, populations may be affected by different kinds of infectious diseases at the same time. Therefore, it is of great significance to study the epidemic model with multiple diseases [39–41].

Recently, Meng et al. [39] considered a novel nonlinear stochastic SIS epidemic model with double epidemic hypothesis as follows:

2 $$ \textstyle\begin{cases} dS= (A-uS(t)-\frac{\beta_{1}S(t)I_{1}(t)}{a_{1}+I_{1}(t)}-\frac{\beta _{2}S(t)I_{2}(t)}{a_{2}+I_{2}(t)}+r_{1}I_{1}(t)+r_{2}I_{2}(t) )\,dt \\ \hphantom{dS={}}{}-\frac{\sigma_{1}S(t)I_{1}(t)}{a_{1}+I_{1}(t)}\,dB_{1}(t)-\frac{\sigma _{2}S(t)I_{2}(t)}{a_{2}+I_{2}(t)}\,dB_{2}(t), \\ dI_{1}= (\frac{\beta_{1}S(t)I_{1}(t)}{a_{1}+I_{1}(t)}-(u+\alpha _{1}+r_{1})I_{1}(t) )\,dt+\frac{\sigma_{1}S(t)I_{1}(t)}{a_{1}+I_{1}(t)}\,dB_{1}(t), \\ dI_{2}= (\frac{\beta_{2}S(t)I_{2}(t)}{a_{2}+I_{2}(t)}-(u+\alpha _{2}+r_{2})I_{2}(t) )\,dt+\frac{\sigma_{2}S(t)I_{2}(t)}{a_{2}+I_{2}(t)}\,dB_{2}(t). \end{cases} $$

They obtained the threshold of system (2) for the extinction and the persistence in mean of the epidemic diseases. Based on system (2), recently, Zhang et al. [40] proposed an SIS system with double epidemic diseases driven by Lévy jumps as follows:

3 $$ \textstyle\begin{cases} dS= (A-uS(t)-\frac{\beta_{1}S(t)I_{1}(t)}{a_{1}+I_{1}(t)}-\frac{\beta _{2}S(t)I_{2}(t)}{a_{2}+I_{2}(t)}+r_{1}I_{1}(t)+r_{2}I_{2}(t) )\,dt \\ \hphantom{dS={}}{}+\sigma_{1}S(t)\,dB_{1}(t)+\int_{\mathbb{Z}}\gamma_{1}(u)S(t^{-})\widetilde {N}(dt,du), \\ dI_{1}= (\frac{\beta_{1}S(t)I_{1}(t)}{a_{1}+I_{1}(t)}-(u+\alpha _{1}+r_{1})I_{1}(t) )\,dt \\ \hphantom{dI_{1}={}}{}+\sigma_{2}I_{1}(t)\,dB_{2}(t)+\int_{\mathbb{Z}}\gamma _{2}(u)I_{1}(t^{-})\widetilde{N}(dt,du), \\ dI_{2}= (\frac{\beta_{2}S(t)I_{2}(t)}{a_{2}+I_{2}(t)}-(u+\alpha _{2}+r_{2})I_{2}(t) )\,dt \\ \hphantom{dI_{2}={}}{}+\sigma_{3}I_{2}(t)\,dB_{3}(t)+\int_{\mathbb{Z}}\gamma _{3}(u)I_{2}(t^{-})\widetilde{N}(dt,du). \end{cases} $$

In model (3), the authors discussed in detail the conditions for persistence in mean and extinction of each epidemic disease. Therefore, they discussed the persistence in mean of susceptible individuals under different conditions. The above two studies provide a theoretical basis for the study of infectious diseases. But they just discussed the persistence in mean and extinction of epidemic diseases under different conditions. In real life, however, when an epidemic outbreak occurs, we do not sit idly but take measures to control the spread of the epidemic disease. There are many ways to suppress the spread of a disease, for instance, cut off transmission routes, pay attention to food hygiene, vaccination and so on [42, 43]. Vaccination is an effective method of preventing infectious diseases and many scientists have explored the effect of vaccination on diseases [44–47].

Motivated by the above works, in this paper, we propose a stochastic SIVS model with double epidemic diseases and Lévy jumps under vaccination as follows:

4 $$ \textstyle\begin{cases} dS= ((1-q)\Lambda-(u+p)S(t)-\frac{\beta_{1}S(t)I_{1}(t)}{\alpha _{1}+I_{1}(t)}-\frac{\beta_{2}S(t)I_{2}(t)}{\alpha _{2}+I_{2}(t)} \\ \hphantom{dS= {}}{}+r_{1}I_{1}(t)+r_{2}I_{2}(t)+\delta V(t) )\,dt \\ \hphantom{dS= {}}{}+\sigma_{3}S\,dB_{3}(t)+\int_{\mathbb{Z}}\gamma_{3}(u)S(t^{-})\widetilde {N}(dt,du), \\ dI_{1}= (\frac{\beta_{1}S(t)I_{1}(t)}{\alpha _{1}+I_{1}(t)}-(u+d_{1}+r_{1})I_{1}(t) )\,dt \\ \hphantom{dI_{1}={}}{}+\sigma_{1}I_{1}\,dB_{1}(t)+\int_{\mathbb{Z}}\gamma_{1}(u)I_{1}(t^{-})\widetilde{N}(dt,du), \\ dI_{2}= (\frac{\beta_{2}S(t)I_{2}(t)}{\alpha _{2}+I_{2}(t)}-(u+d_{2}+r_{2})I_{2}(t) )\,dt \\ \hphantom{dI_{2}={}}{}+\sigma_{2}I_{2}\,dB_{2}(t)+\int_{\mathbb{Z}}\gamma_{2}(u)I_{2}(t^{-})\widetilde{N}(dt,du), \\ dV= [q\Lambda+pS(t)-(u+\delta)V(t) ]\,dt+\sigma_{4}V\,dB_{4}(t) \\ \hphantom{dV={}}{}+\int _{\mathbb{Z}}\gamma_{4}(u)V(t^{-})\widetilde{N}(dt,du), \end{cases} $$

where $S(t)$, $I_{1}(t)$, $I_{2}(t)$, $V(t)$, respectively, stand for the density of susceptible, infective *A*, infective *B* and vaccinated individuals at time *t*, Λ is a constant input of new numbers into the population, *q* means a fraction of vaccinated for the newborn, $\beta _{i}$ is the infection rate coefficient from $I_{i}(t)$ ($i=1,2$) to $S(t)$, respectively. *u* represents the natural death rate of $S(t)$, $I_{1}(t)$, $I_{2}(t)$, $V(t)$, *p* is the proportional coefficient of vaccinated for the susceptible, $r_{i}$, $d_{i}$ is the recovery rate and disease-caused death rate of $I_{i}(t)$, $i=1,2$, respectively. *δ* stands for the rate of losing their immunity for vaccinated individuals, $\alpha_{1}$ and $\alpha_{2}$ are the so-called half-saturation constants, respectively. $B(t)=(B_{1}(t),B_{2}(t),B_{3}(t),B_{4}(t))$ is a standard Brownian motion with intensity $\sigma_{i}>0$ ($i=1,2,3,4$).

Throughout this paper, let $(\Omega,\mathcal{F},\{\mathcal{F}\}_{t\geq 0},\mathcal{P})$ be a complete probability space with a filtration $\{\mathcal{F}_{t}\} _{t\geq0}$ satisfying the usual conditions (i.e. it is increasing and right continuous while $\mathcal{F}_{0}$ contains all $\mathcal{P}$-null sets). Function $B_{i}(t)$ ($i=1,2,3,4$) is a Brownian motion defined on the complete probability space Ω, the intensity of $B_{i}(t)$ is $\sigma_{i}$ ($i=1,2,3,4$). $\widetilde{N}(dt,du)=N(dt,du)-\lambda(du)\,dt$, *N* is a Poisson counting measure on $(0,+\infty)\times\mathbb{Z}$, *λ* is the characteristic measure of *N* on a measurable subset $\mathbb{Z}$, $\lambda(\mathbb{Z})<+\infty$, $\gamma_{i}$ ($i=1,2,3,4$) is bounded and continuous with respect to *λ* and is $\mathcal {B}(\mathbb{Z})\times\mathcal{F}_{t}$-measurable. For an integrable function $X(t)$ on $[0,+\infty)$, we define $\langle X(t)\rangle=\frac {1}{t}\int^{t}_{0}X(s)\,ds$.

The main purpose of this paper is to investigate the threshold dynamics of the stochastic SIVS epidemic model. In this paper, by using the Lyapunov method and the technique of a series of stochastic inequalities, we obtain sufficient conditions for the persistence in mean and extinction of the stochastic system and the threshold which governs the extinction and the spread of the epidemic diseases. Our results significantly improve and generalize the corresponding results in recent literatures. The developed theoretical methods and stochastic inequalities technique can be used to investigate the high-dimensional nonlinear stochastic differential systems. In Section 2, we firstly give some lemmas and recall some necessary notations and definitions. Furthermore, we obtain the main results for stochastic disease-free dynamics and stochastic endemic dynamics which imply the extinction and the spread of the epidemic diseases. Finally, this paper gives the conclusions and numerical simulations investigating the dynamical effects of stochastic disturbance.

## Main results

The main purpose of this paper is to investigate the threshold dynamics of the stochastic SIVS epidemic model. In this section, by using the technique of a series of stochastic inequalities, we obtain sufficient conditions for the persistence in mean and extinction of the stochastic system and the threshold which governs the extinction and the spread of epidemic diseases.

### Preliminary knowledge

For the sake of notational simplicity, we define

$$\begin{aligned}& b_{i}=\frac{1}{2}\sigma_{i}^{2}+ \int_{\mathbb{Z}}\bigl[\gamma_{i}(u)-\ln\bigl(1+\gamma _{i}(u)\bigr)\bigr]\lambda(du),\quad i=1,2,3,4; \\& R_{i}=\frac{\beta_{i}(u+\delta-uq)\Lambda}{u^{2}+u\delta+up}-\alpha_{i} (u+d_{i}+r_{i}+b_{i} ),\quad i=1,2; \\& \check{\gamma}(u)=\max\bigl\{ \gamma_{1}(u),\gamma_{2}(u), \gamma_{3}(u),\gamma _{4}(u)\bigr\} ; \\& \hat{\gamma}(u)=\min\bigl\{ \gamma_{1}(u),\gamma_{2}(u), \gamma_{3}(u),\gamma_{4}(u)\bigr\} ; \\& \phi= \int_{\mathbb{Z}}\bigl[\bigl(1+\check{\gamma}(u) \bigr)^{\varrho}-1-\hat{\gamma }(u)\bigr]v(du); \\& \sigma=\max\{\sigma_{1},\sigma_{2},\sigma_{3}, \sigma_{4}\}. \end{aligned}$$

Throughout this paper, suppose that the following two assumptions hold.

#### Assumption 2.1

The following hold:

(i) $1+\gamma_{i}(u)>0$;
(ii) $\int_{\mathbb{Z}} [\gamma_{i}(u)-\ln(1+\gamma_{i}(u)) ]\lambda(du)<\infty$, $i=1,2,3,4$, $u\in\mathbb{Z}$.

#### Remark 2.1

This assumption means that the intensities of Lévy noises are not infinite.

#### Assumption 2.2

Suppose that there exists some $\varrho>1$ such that the following inequality holds:

$$b=u-\frac{\varrho-1}{2}\sigma^{2}-\frac{\phi}{\varrho}>0. $$

#### Definition 2.1

[39]

(i) The species $X(t)$ is said to be extinctive if $\lim_{t\rightarrow+\infty}X(t)=0$;
(ii) The species $X(t)$ is said to be persistent in mean if $\lim_{t\rightarrow+\infty}\langle X(t)\rangle_{*}>0$.

The following elementary inequality will be used frequently in the sequel.

#### Lemma 2.1

Burkholder-Davis-Gundy inequality [48]

*Let* $g\in\mathcal {L}^{2}(R_{+};R^{d\times m})$. *For any* $t\geq0$, *define*

$$x(t)= \int^{t}_{0}g(s)\,dB(s),\qquad A(t)= \int_{t}^{0} \bigl\vert g(s) \bigr\vert ^{2}\,ds. $$

*Then*, *for every* $p>0$, *there exist two positive constants* $c_{p}$, $C_{p}$ *such that*

$$c_{p}E \bigl\vert A(t) \bigr\vert ^{p/2}\leq E \Bigl(\sup _{0\leq s\leq t \vert x(s) \vert ^{p}} \Bigr)\leq C_{p}E \bigl\vert A(t) \bigr\vert ^{p/2},\quad t\geq0, $$

*where* $c_{p}$, $C_{p}$ *only depend on* *p*.

#### Lemma 2.2

Chebyshev inequality [48]

*For any* $c>0$, $p>0$, $X\in L^{p}$, *the following inequality holds*:

$$P\bigl\{ w: \bigl\vert X(w) \bigr\vert \geq c\bigr\} \leq c^{-p}E \vert X \vert ^{p}. $$

#### Lemma 2.3

Hölder inequality [48]

*For any* $a_{i},b_{i}\in R$ *and* $k\geq2$, *if* $p,q>1$ *and* $\frac{1}{p}+\frac{1}{q}=1$, *the following inequality holds*:

$$\Biggl\vert \sum_{i=1}^{k}a_{i}b_{i} \Biggr\vert \leq \Biggl(\sum_{i=1}^{k}|a_{i}|^{p} \Biggr)^{1/p} \Biggl(\sum_{i=1}^{k}|b_{i}|^{q} \Biggr)^{1/q}. $$

#### Lemma 2.4

Doob’s martingale inequality [48]

*Let* *X* *be a submartingale taking nonnegative real values*, *either in discrete or continuous time*. *That is*, *for all times* *s* *and* *t* *with* $s< t$,

$$X_{s}\leq E[X_{t}|\mathcal{F}_{s}]. $$

*Then*, *for any constant* $C>0$,

$$P \Bigl[\sup_{0\leq t\leq T}X_{t}\geq C \Bigr]\leq \frac{E[|X_{T}|]}{C}, $$

*where* *P* *denotes the probability measure on the sample space* Ω *of the stochastic process* $X: [0,T]\times\Omega\rightarrow[0,+\infty)$ *and* *E* *denotes the expected value with respect to the probability measure* *P*.

#### Lemma 2.5

[49, 50]

*Assume that* $X(t)\in R^{+}$ *is an Itô’s*-*Lévy process of the form*

$$dX(t)=F\bigl(X\bigl(t^{-}\bigr),t^{-}\bigr)\,dt+G\bigl(X\bigl(t^{-}\bigr),t^{-}\bigr) \,dB(t)+ \int_{\mathbb{Z}}H\bigl(X\bigl(t^{-}\bigr),t^{-},u\bigr) \widetilde{N}(dt,du), $$

*where* $F : R^{n}\times R_{+}\times S\rightarrow R^{n}$, $G : R^{n}\times R_{+}\times S\rightarrow R^{n}$ *and* $H : R^{n}\times R_{+}\times S\times Z\rightarrow R^{n}$ *are measurable functions*.

*Given* $V\in C^{2,1}(R^{n}\times R_{+}\times S; R_{+})$, *we define the operator* *LV* *by*

$$\begin{aligned} \mathit{LV}(X,t) =&V_{t}(X,t)+V_{X}(X,t)F(X,t)+ \frac{1}{2}\operatorname{trace} \bigl[G^{T}(X,t)V_{XX}(X,t)G(X,t) \bigr] \\ &{}+ \int_{\mathbb{Z}} \bigl\{ V\bigl(X+H(X,t)\bigr)-V(X,t)-V_{X}(X,t)H(X,t,u) \bigr\} \lambda(du), \end{aligned}$$

*where*

$$\begin{aligned}& V_{t}(X,t)=\frac{\partial V_{X}(X,t)}{\partial t}, \\& V_{X}(X,t)= \biggl( \frac {\partial V_{X}(X,t)}{\partial X_{1}},\ldots,\frac{\partial V_{X}(X,t)}{\partial X_{n}} \biggr), \\& V_{XX}(X,t)= \biggl(\frac{\partial^{2} V_{X}(X,t)}{\partial X_{i}\partial X_{j}} \biggr)_{n\times n}. \end{aligned}$$

*Then the generalized Itô’s formula with Lévy jumps is given by*

$$ dV(X,t)= \mathit{LV}(X,t)\,dt+V_{X}(X,t)G(X,t)\,dB(t)+ \int_{\mathbb{Z}} \bigl\{ V\bigl(X+H(X,t)\bigr)-V(X,t) \bigr\} \widetilde{N}(dt,du). $$

#### Lemma 2.6

[51]

*Let* $X(t)\in C(\Omega\times[0,+\infty),R_{+})$. *We have the following conclusions*.

(i) *If there exist* $T>0$, $\lambda_{0}>0$, *λ*, *m*, $n_{i}$ *such that when* $t\geq T$,
  $$\ln X(t)\leq\lambda t-\lambda_{0} \int_{0}^{t}X(s)\,ds+mB(t)+\sum _{i=1}^{j}n_{i} \int _{0}^{t} \int_{\mathbb{Z}}\ln\bigl(1+\gamma_{i}(u)\bigr) \widetilde{\Gamma}(ds,du)\quad \textit{a.s.}, $$
  *then*
  $$ \textstyle\begin{cases} \langle X\rangle^{*}\leq\frac{\lambda}{\lambda_{0}}\quad \textit{a.s., if } \lambda\geq 0;\\ \lim_{t\rightarrow+\infty}X(t)=0 \quad \textit{a.s., if } \lambda< 0. \end{cases} $$
(ii) *If there exist* $T>0$, $\lambda_{0}>0$, $\lambda>0$, *m*, $n_{i}$ *such that when* $t\geq T$,
  $$\ln X(t)\geq\lambda t-\lambda_{0} \int_{0}^{t}X(s)\,ds+mB(t)+\sum _{i=1}^{j}n_{i} \int _{0}^{t} \int_{\mathbb{Z}}\ln\bigl(1+\gamma_{i}(u)\bigr) \widetilde{\Gamma}(ds,du) \quad \textit{a.s.}, $$
  *then* $\langle X\rangle_{*}\geq\frac{\lambda}{\lambda_{0}}$ *a*.*s*.

#### Lemma 2.7

*For any initial value* $(S(0),I_{1}(0),I_{2}(0),V(0))\in R_{+}^{4}$, *the solution* $(S(t),I_{1}(t),I_{2}(t), V(t))$ *of model* (4) *has the following property*:

$$\lim_{t\rightarrow\infty}\frac{S(t)+I_{1}(t)+I_{2}(t)+V(t)}{t}=0\quad \textit{a.s.} $$

*Moreover*,

$$\begin{aligned}& \lim_{t\rightarrow\infty}\frac{S(t)}{t}=0,\qquad \lim _{t\rightarrow\infty }\frac{I_{1}(t)}{t}=0, \\& \lim_{t\rightarrow\infty} \frac{I_{2}(t)}{t}=0,\qquad \lim_{t\rightarrow\infty}\frac{V(t)}{t}=0\quad \textit{a.s.} \\& \lim_{t\rightarrow\infty}\frac{\ln S(t)}{t}\leq0,\qquad \lim _{t\rightarrow \infty}\frac{\ln I_{1}(t)}{t}\leq0, \\& \lim_{t\rightarrow\infty} \frac{\ln I_{2}(t)}{t}\leq0,\qquad \lim_{t\rightarrow\infty}\frac{\ln V(t)}{t}\leq0 \quad \textit{a.s.} \end{aligned}$$

#### Proof

Define

$$X=S+I_{1}+I_{2}+V,\qquad Q(X)=X^{\varrho}. $$

Applying the generalized Itô’s formula to $Q(X)$, we have

5 $$\begin{aligned} dQ(X) \leq&LQdt+\varrho X^{\varrho-1} \bigl(\sigma_{1}I_{1} \,dB_{1}(t)+\sigma _{2}I_{2}\,dB_{2}(t)+ \sigma_{3}S\,dB_{3}(t)+\sigma_{4}V \,dB_{4}(t) \bigr) \\ &{}+X^{\varrho}\int_{\mathbb{Z}}\bigl[\bigl(1+\check{\gamma}(u) \bigr)^{\varrho}-\hat{\gamma}\bigr]\widetilde{N}(dt,du), \end{aligned}$$

where

$$\begin{aligned} LQ \leq&\varrho X^{\varrho-1}(\Lambda-uX-d_{1}I_{1}-d_{2}I_{2})+ \frac{\varrho (\varrho-1)}{2}X^{\varrho-2}\sigma^{2}X^{2}+\phi X^{\varrho}\\ \leq&\varrho X^{\varrho-2} \biggl[\Lambda X- \biggl(u-\frac{\varrho -1}{2} \sigma^{2}-\frac{\phi}{\varrho} \biggr)X^{2} \biggr]. \end{aligned}$$

Choose a positive constant $\varrho>1$ that satisfies

$$b=u-\frac{\varrho-1}{2}\sigma^{2}-\frac{\phi}{\varrho}>0. $$

For any constant *k* satisfying $k\in(0,b\varrho)$, one has

$$\begin{aligned} de^{kt}Q\bigl(X(t)\bigr) \leq&L\bigl[e^{kt}Q \bigl(X(t)\bigr)\bigr]\,dt+e^{kt}\varrho X^{\varrho-1}\bigl[\sigma _{1}I_{1}(s)\,dB_{1}(s)+\sigma_{2}I_{2}(s) \,dB_{2}(s) \\ &{}+\sigma_{3}S(s)\,dB_{3}(s)+\sigma_{4}V(s) \,dB_{4}(s)\bigr] \\ &{}+e^{kt}\varrho X^{\varrho}\int _{\mathbb{Z}}\bigl[\bigl(1+\check{\gamma}(u) \bigr)^{\varrho}-\hat{\gamma}\bigr]\widetilde{N}(dt,du). \end{aligned}$$

Integrating from 0 to *t* and taking expectation on both sides of (5), we have

$$ \mathbb{E}e^{kt}Q\bigl(X(t)\bigr)\leq Q\bigl(X(0)\bigr)+\mathbb{E} \biggl[ \int _{0}^{t}\bigl[ke^{ks}Q\bigl(X(s) \bigr)+e^{ks}LQ\bigl(X(s)\bigr)\bigr]\,ds \biggr]. $$

Easily, one has

$$\begin{aligned} ke^{kt}Q\bigl(X(t)\bigr)+e^{kt}LQ\bigl(X(t)\bigr) \leq&ke^{kt}X^{\varrho}(t)+\varrho e^{kt}X^{\varrho-2}(t) \bigl[-bX^{2}(t)+\Lambda X(t)\bigr] \\ \leq&\varrho e^{kt}\sup_{X\in R^{+}} \biggl\{ X^{\varrho-2} \biggl[- \biggl(b-\frac{k}{\varrho} \biggr)X^{2}+ \Lambda X \biggr]+1 \biggr\} \\ :=&\varrho e^{kt}H. \end{aligned}$$

Therefore

6 $$ \mathbb{E}\bigl(X^{\varrho}\bigr)\leq\frac{X^{\varrho}(0)}{e^{kt}}+ \frac{\varrho H}{k}\leq X^{\varrho}(0)+\varrho H:=M. $$

By Lemma 2.1, applying the Burkholder-Davis-Gundy inequality, integrating equation (5) from 0 to *t*, and for an arbitrarily small positive constant *δ*, one has

$$\begin{aligned} \mathbb{E}\Bigl[\sup_{k\delta\leq t\leq(k+1)\delta}\bigl(X^{\varrho}(t)\bigr) \Bigr] \leq &E\bigl(X(k\delta)\bigr)^{\varrho}+Y_{1}+Y_{2} \\ \leq&M+Y_{1}+Y_{2}, \end{aligned}$$

where

$$\begin{aligned} Y_{1} =&\mathbb{E} \biggl\{ \sup_{k\delta\leq t\leq(k+1)\delta} \biggl\vert \int _{k\delta}^{t}\varrho X^{\varrho-2}(s) \bigl[-bX^{2}(s)+\Lambda X(s)\bigr]\,ds \biggr\vert \biggr\} \\ \leq& c_{\varrho}\mathbb{E} \biggl[\sup_{k\delta\leq t\leq (k+1)\delta} \biggl\vert \int_{k\delta}^{t} X^{\varrho}(s)\,ds \biggr\vert \biggr] \\ \leq&c_{\varrho}\mathbb{E} \biggl[ \int_{k\delta}^{(k+1)\delta}X^{\varrho }(s)\,ds \biggr]\leq c_{\varrho}\delta\mathbb{E} \Bigl[\sup_{k\delta\leq t\leq (k+1)\delta}X^{\varrho}(s) \,ds \Bigr], \quad k=1,2,\ldots \end{aligned}$$

and

$$\begin{aligned} Y_{2} =&\mathbb{E} \biggl\{ \sup_{k\delta\leq t\leq(k+1)\delta} \biggl| \int _{k\delta}^{t}\varrho X^{\varrho-1}(s)\bigl[ \sigma_{1}I_{1}(s)\,dB_{1}(s)+\sigma _{2}I_{2}(s)\,dB_{2}(s)+\sigma_{3}S(s) \,dB_{3}(s) \\ &{}+\sigma_{4}V(s)\,dB_{4}(s)\bigr]+ \int_{k\delta}^{t}X^{\varrho}(s) \int_{\mathbb{Z}}\bigl[\bigl(1+\check{\gamma}(u) \bigr)^{\varrho}-\hat{\gamma}\bigr]\widetilde {N}(dt,du) \biggr| \biggr\} \\ \leq&C_{\varrho}\mathbb{E} \biggl[ \int_{k\delta}^{(k+1)\delta}\varrho ^{2}X^{2(\varrho-1)} \bigl(\sigma_{1}^{2}I_{1}^{2}+ \sigma_{2}^{2}I_{2}^{2}+ \sigma_{3}^{2}S^{2}+\sigma _{4}^{2}V^{2} \bigr)\,ds \biggr]^{\frac{1}{2}} \\ &{}+C_{\varrho}E \biggl\{ \int_{k\delta}^{(k+1)\delta}X^{2\varrho} \int_{\mathbb{Z}}\bigl[\bigl(1+\check{\gamma}(u) \bigr)^{\varrho}-\hat{\gamma}(u)\bigr]^{2}v(du)\,ds \biggr\} ^{\frac{1}{2}} \\ \leq&C_{\varrho}\delta^{\frac{1}{2}} \biggl[\varrho\sigma+ \int_{\mathbb{Z}}\bigl[\bigl(1+\check{\gamma}(u) \bigr)^{\varrho}-\hat{\gamma}(u)\bigr]^{2}v(du) \biggr]\mathbb {E} \Bigl[\sup_{k\delta\leq t\leq(k+1)\delta}X^{\varrho}\Bigr], \quad k=1,2,\ldots, \end{aligned}$$

where $c_{\varrho}, C_{\varrho}>0$.

So we have

$$\begin{aligned} \mathbb{E}\Bigl[\sup_{k\delta\leq t\leq(k+1)\delta}\bigl(X^{\varrho}(t)\bigr) \Bigr] \leq &E\bigl(X(k\delta)\bigr)^{\varrho}+c_{\varrho}\delta \mathbb{E}\Bigl[\sup_{k\delta\leq t\leq (k+1)\delta}X^{\varrho}(s)\,ds\Bigr] \\ &{}+C_{\varrho}\delta^{\frac{1}{2}} \biggl[\varrho\sigma+ \int_{\mathbb{Z}}\bigl[\bigl(1+\check{\gamma}(u) \bigr)^{\varrho}-\hat{\gamma}(u)\bigr]^{2}v(du) \biggr] \\ &{}\times\mathbb {E} \Bigl[\sup_{k\delta\leq t\leq(k+1)\delta}X^{\varrho}\Bigr]. \end{aligned}$$

Choose a positive constant *δ* that satisfies

$$ c_{\varrho}\delta+C_{\varrho}\delta^{\frac{1}{2}} \biggl[\varrho \sigma+ \int _{\mathbb{Z}}\bigl[\bigl(1+\check{\gamma}(u) \bigr)^{\varrho}-\hat{\gamma}(u)\bigr]^{2}v(du) \biggr]\leq \frac{1}{2}. $$

Combining it with equation (6), one has

$$ \mathbb{E}\Bigl[\sup_{k\delta\leq t\leq(k+1)\delta}\bigl(X^{\varrho}(t)\bigr) \Bigr]\leq 2\mathbb{E}\bigl(X(k\delta)\bigr)^{\varrho}\leq2M. $$

Applying the arbitrariness of $\kappa_{X}>0$ and Lemma 2.2 for Chebyshev’s inequality, one obtains

$$\begin{aligned} \begin{aligned} \mathbb{P} \Bigl\{ \sup_{k\delta\leq t\leq(k+1)\delta}X^{\varrho}(t)>(k \delta)^{1+\kappa X} \Bigr\} &\leq\frac{\mathbb{E}[\sup_{k\delta \leq t\leq(k+1)\delta}X^{\varrho}(t)]}{(k\delta)^{1+\kappa X}} \\ &\leq\frac{2M}{(k\delta)^{1+\kappa X}}, \quad k=1,2,\ldots. \end{aligned} \end{aligned}$$

Applying the Borel-Cantelli lemma [48], for almost all $\omega\in\Omega$, one has

7 $$ \sup_{k\delta\leq t\leq(k+1)\delta}X^{\varrho}(t)\leq(k \delta)^{1+\kappa X} $$

holds for all but finitely many *k*. Therefore, for any positive constant $k\geq k_{0}$ and almost all $\omega\in\Omega$, there is $k_{0}(\omega)$ such that equation (7) holds.

Thus, for almost all $\omega\in\Omega$, once conditions $k\geq k_{0}$ and $k\delta\leq t\leq(k+1)\delta$ hold, then we have

8 $$ \frac{\ln X^{\varrho}(t)}{\ln t}\leq\frac{(1+\kappa_{X})\ln(k\delta)}{\ln (k\delta)}=1+\kappa_{X}. $$

Taking the limit superior on both sides of equation (8) and applying the arbitrariness of $\kappa_{X}>0$, one has

$$ \limsup_{t\rightarrow\infty}\frac{\ln X^{\varrho}(t)}{\ln t}\leq1 \quad \mbox{a.s.} $$

Easily, for any *ϱ* satisfying $1<\varrho<1+\frac{2(u-\phi )}{\sigma^{2}}$, one has $u>\frac{\varrho-1}{2}\sigma^{2}+\phi$. Therefore

$$ \limsup_{t\rightarrow\infty}\frac{\ln X^{\varrho}(t)}{\ln t}\leq\frac {1}{\varrho}\quad \mbox{a.s.} $$

That is to say, for any constant *τ* satisfying $0<\tau<1-\frac {1}{\varrho}$, there is a constant $N=N(\omega)$, and once condition $t\geq N$ holds, then we have

$$ \ln X(t)\leq \biggl(\frac{1}{\varrho}+\tau \biggr)\ln t. $$

Therefore

$$ \lim_{t\rightarrow\infty}\frac{X(t)}{t}=\lim_{t\rightarrow\infty} \frac {S(t)+I_{1}(t)+I_{2}(t)+V(t)}{t}=0\leq\limsup_{t\rightarrow\infty}\frac {t^{\frac{1}{\varrho}+\tau}}{t}=0 \quad \mbox{a.s.} $$

So

$$\begin{aligned}& \lim_{t\rightarrow\infty}\frac{S(t)}{t}=0,\qquad \lim _{t\rightarrow\infty }\frac{I_{1}(t)}{t}=0, \\& \lim_{t\rightarrow\infty} \frac{I_{2}(t)}{t}=0,\qquad \lim_{t\rightarrow\infty}\frac{V(t)}{t}=0\quad \mbox{a.s.} \end{aligned}$$

and

$$\begin{aligned}& \lim_{t\rightarrow\infty}\frac{\ln S(t)}{t}\leq0,\qquad \lim _{t\rightarrow \infty}\frac{\ln I_{1}(t)}{t}\leq0, \\& \lim_{t\rightarrow\infty} \frac{\ln I_{2}(t)}{t}\leq0,\qquad \lim_{t\rightarrow\infty}\frac{\ln V(t)}{t}\leq0 \quad \mbox{a.s.} \end{aligned}$$

This completes the proof. □

#### Lemma 2.8

*For any initial value* $(S(0),I_{1}(0),I_{2}(0),V(0))\in R_{+}^{4}$, *the solution* $(S(t),I_{1}(t),I_{2}(t), V(t))$ *of model* (4) *has the following property*:

$$\begin{aligned}& \lim_{t\rightarrow\infty}\frac{\int_{0}^{t}I_{1}(s)\,dB_{1}(s)}{t}=0,\qquad \lim _{t\rightarrow\infty}\frac{\int_{0}^{t}\int_{\mathbb{Z}}\gamma _{1}(u)I_{1}(s)\widetilde{N}(ds,du)}{t}=0\quad \textit{a.s.}, \\& \lim_{t\rightarrow\infty}\frac{\int_{0}^{t}I_{2}(s)\,dB_{2}(s)}{t}=0,\qquad \lim _{t\rightarrow\infty}\frac{\int_{0}^{t}\int_{\mathbb{Z}}\gamma _{2}(u)I_{2}(s)\widetilde{N}(ds,du)}{t}=0\quad \textit{a.s.}, \\& \lim_{t\rightarrow\infty}\frac{\int_{0}^{t}S(s)\,dB_{3}(s)}{t}=0,\qquad \lim _{t\rightarrow\infty}\frac{\int_{0}^{t}\int_{\mathbb{Z}}\gamma _{3}(u)S(s)\widetilde{N}(ds,du)}{t}=0\quad \textit{a.s.}, \\& \lim_{t\rightarrow\infty}\frac{\int_{0}^{t}V(s)\,dB_{4}(s)}{t}=0,\qquad \lim _{t\rightarrow\infty}\frac{\int_{0}^{t}\int_{\mathbb{Z}}\gamma _{4}(u)V(s)\widetilde{N}(ds,du)}{t}=0\quad \textit{a.s.} \end{aligned}$$

#### Proof

Define

$$\begin{aligned}& X_{1}(t)= \int_{0}^{t}I_{1}(s)\,dB_{1}(s), \qquad Y_{1}(t)= \int_{0}^{t} \int_{\mathbb{Z}}\gamma _{1}(u)I_{1}(s) \widetilde{N}(ds,du), \\& X_{2}(t)= \int_{0}^{t}I_{2}(s)\,dB_{2}(s), \qquad Y_{2}(t)= \int_{0}^{t} \int_{\mathbb{Z}}\gamma _{2}(u)I_{2}(s) \widetilde{N}(ds,du), \\& X_{3}(t)= \int_{0}^{t}S(s)\,dB_{3}(s),\qquad Y_{3}(t)= \int_{0}^{t} \int_{\mathbb{Z}}\gamma _{3}(u)S(s)\widetilde{N}(ds,du), \\& X_{4}(t)= \int_{0}^{t}V(s)\,dB_{4}(s),\qquad Y_{4}(t)= \int_{0}^{t} \int_{\mathbb{Z}}\gamma _{4}(u)V(s)\widetilde{N}(ds,du). \end{aligned}$$

Applying Lemma 2.1 for the Burkholder-Davis-Gundy inequality and Lemma 2.3 for Hölder’s inequality, one has

$$\begin{aligned}& \mathbb{E} \Bigl[\sup_{0\leq s\leq t} \bigl\vert X_{1}(s) \bigr\vert ^{\varrho}\Bigr]\leq C_{\varrho}\mathbb{E} \biggl[ \int_{0}^{t}I_{1}^{2}(\theta)\,d \theta \biggr]^{\frac {\varrho}{2}}\leq C_{\varrho}\mathbb{E} \biggl[ \int_{0}^{t} \bigl\vert I_{1}^{2}( \theta ) \bigr\vert \,d\theta \biggr]^{\frac{\varrho}{2}}, \\& \mathbb{E} \Bigl[\sup_{0\leq s\leq t} \bigl\vert Y_{1}(s) \bigr\vert ^{\varrho}\Bigr]\leq C_{\varrho}\mathbb{E} \biggl[ \int_{0}^{t} \int_{\mathbb{Z}}I_{1}^{2}(\theta)\gamma _{1}^{2}(u)\,d\theta \biggr]^{\frac{\varrho}{2}} \\& \hphantom{\mathbb{E} \Bigl[\sup_{0\leq s\leq t} \bigl\vert Y_{1}(s) \bigr\vert ^{\varrho}\Bigr]}\leq C_{\varrho}\biggl( \int _{\mathbb{Z}}\gamma_{1}^{2}(u)v(du) \biggr)^{\frac{\varrho}{2}}\mathbb{E} \biggl[ \int_{0}^{t} \bigl\vert I_{1}(\theta) \bigr\vert \,d\theta \biggr]^{\frac{\varrho}{2}} \end{aligned}$$

for $2<\varrho<1+\frac{2(u-\phi)}{\sigma^{2}}$. Here $C_{\varrho}= [\frac{\varrho^{\varrho+1}}{2(\varrho-1)^{\varrho-1}} ]^{\frac {\varrho}{2}}>0$ is a constant.

Applying equation (6), we have

$$\mathbb{E} \Bigl[\sup_{k\leq t\leq(k+1)}\bigl|X_{1}(s)\bigr|^{\varrho}\Bigr]\leq 2MC_{\varrho}(k +1)^{\frac{\varrho}{2}}\leq2^{1+\frac{\varrho }{2}}MC_{\varrho}k^{\frac{\varrho}{2}}. $$

For any constant $\kappa_{X_{1}}>0$, applying Lemma 2.4 for Doob’s martingale inequality, one obtains

$$\begin{aligned} \mathbb{P} \Bigl\{ \omega:\sup_{k\leq t\leq(k+1)} \bigl\vert X_{1}(t) \bigr\vert ^{\varrho}>k^{1+\kappa_{X_{1}}+\frac{\varrho}{2}} \Bigr\} \leq& \frac{\mathbb{E}[ {\sup_{k\leq t\leq(k+1)}} \vert X_{1}(k+1) \vert ^{\varrho}]}{k^{1+\kappa_{X_{1}}+\frac {\varrho}{2}}}\leq\frac{2^{1+\frac{\varrho}{2}}MC_{\varrho}k^{\frac {\varrho}{2}}}{k^{1+\kappa_{X_{1}}+\frac{\varrho}{2}}} \\ \leq&\frac{2^{1+\frac{\varrho}{2}}MC_{\varrho}}{k^{1+\kappa_{X_{1}}}}, \quad k=1,2,\ldots. \end{aligned}$$

Applying the Borel-Cantelli lemma, one has

9 $$ \frac{\ln|X_{1}(t)|^{\varrho}}{\ln t}\leq\frac{(1+\kappa_{X_{1}}+\frac{\varrho }{2})\ln k}{\ln k}=1+\kappa_{X_{1}}+ \frac{\varrho}{2}. $$

Taking the limit superior on both sides of equation (9) and applying the arbitrariness of $\kappa_{X}>0$, one has

$$\limsup_{t\rightarrow\infty}\frac{\ln|X_{1}(t)|}{\ln t}\leq\frac {1}{2}+ \frac{1}{\varrho}\quad \mbox{a.s.} $$

That is to say, for any constant *τ* satisfying $0<\tau<\frac {1}{2}-\frac{1}{\varrho}$, there is a constant $N=N(\omega)$, and once $t\geq N$, $w\in\Omega_{\tau}$ holds, then we have

10 $$ \ln \bigl\vert X_{1}(t) \bigr\vert \leq \biggl( \frac{1}{2}+\frac{1}{\varrho}+\tau \biggr)\ln t. $$

Dividing both sides of equation (10) by *t* and taking the limit superior, we have

$$\limsup_{t\rightarrow\infty}\frac{|X_{1}(t)|}{t}\leq\limsup _{t\rightarrow \infty}\frac{t^{\frac{1}{2}+\frac{1}{\varrho}+\tau}}{t}=0. $$

Combining it with $\liminf_{t\rightarrow\infty}\frac{|X_{1}(t)|}{t}\geq0$, one has

$$\lim_{t\rightarrow\infty}\frac{|X_{1}(t)|}{t}=\lim_{t\rightarrow\infty } \frac{ X_{1}(t)}{t}=0\quad \mbox{a.s.} $$

Similarly, one obtains

$$\begin{aligned}& \lim_{t\rightarrow\infty}\frac{\ln X_{2}(t)}{t}=0,\qquad \lim _{t\rightarrow \infty}\frac{\ln X_{3}(t)}{t}=0, \\& \lim_{t\rightarrow\infty} \frac{\ln X_{4}(t)}{t}=0,\qquad \lim_{t\rightarrow\infty}\frac{\ln Y_{1}(t)}{t}=0, \\& \lim _{t\rightarrow \infty}\frac{\ln Y_{2}(t)}{t}=0,\qquad \lim_{t\rightarrow\infty} \frac{\ln Y_{3}(t)}{t}=0,\qquad \lim_{t\rightarrow\infty}\frac{\ln Y_{4}(t)}{t}=0. \end{aligned}$$

This completes the proof. □

#### Lemma 2.9

*For any initial value* $(S(0),I_{1}(0),I_{2}(t),V(0))\in R_{+}^{4}$, *model* (4) *has a unique positive solution* $(S(t),I_{1}(t),I_{2}(t),V(t))\in R_{+}^{4}$ *on* $t\geq0$ *with probability* 1.

#### Proof

The proof is similar to Refs. [9, 44] by defining $Q(S,I_{1},I_{2},V)=S-1-\ln S+I_{1}-1-\ln I_{1}+I_{2}-1-\ln I_{2}+V-1-\ln V$, and hence is omitted. □

### Stochastic disease-free dynamics

#### Theorem 2.1

*Suppose that conditions* $R_{1}<0$ *and* $R_{2}<0$ *hold*. *Then*, *for any initial value* $(S(0),I_{1}(0),I_{2}(0),V(0))\in R_{+}^{4}$, *the solution* $(S(t),I_{1}(t),I_{2}(t),V(t))$ *of model* (4) *has the following property*:

$$\begin{aligned}& \lim_{t\rightarrow\infty}I_{i}(t)=0,\quad i=1,2,\qquad \lim _{t\rightarrow\infty }\bigl\langle S(t)\bigr\rangle =\frac{(u+\delta-uq)\Lambda}{u^{2}+u\delta+up}, \\& \lim_{t\rightarrow\infty}\bigl\langle V(t)\bigr\rangle =\frac{(p+uq)\Lambda }{u^{2}+u\delta+up}. \end{aligned}$$

*That is to say*, *the two epidemic diseases go to extinct almost surely*.

#### Proof

By equation (4), one has

11 $$\begin{aligned} d \biggl(S+I_{1}+I_{2}+\frac{\delta}{u+\delta}V \biggr) =&\frac{(u+\delta -uq)\Lambda}{u+\delta}-\frac{u^{2}+u\delta+up}{u+\delta}S-\sum _{i=1}^{2}(u+d_{i})I_{i} \\ &{}+\sigma_{3}S\,dB_{3}(t)+ \int_{\mathbb{Z}}\gamma_{3}(u)S\bigl(t^{-}\bigr)\widetilde {N}(dt,du) \\ &{}+\sum_{i=1}^{2} \biggl[ \sigma_{i}I_{i}d B_{i}(t)+ \int_{\mathbb{Z}}\gamma _{i}(u)I_{i}\bigl(t^{-} \bigr)\widetilde{N}(dt,du) \biggr] \\ &{}+\frac{\delta}{u+\delta} \biggl(\sigma_{4}V\,dB_{4}(t)+ \int_{\mathbb{Z}}\gamma _{4}(u)V\bigl(t^{-}\bigr) \widetilde{N}(dt,du) \biggr). \end{aligned}$$

Dividing both sides of equation (11) by *t* and integrating over the time interval 0 to *t* yield

12 $$ \bigl\langle S(t)\bigr\rangle =\frac{u+\delta}{u^{2}+u\delta+up} \Biggl[ \frac{(u+\delta -uq)\Lambda}{u+\delta}-\sum_{i=1}^{2} (u+d_{i} )\bigl\langle I_{i}(t)\bigr\rangle -\Phi(t) \Biggr], $$

where

$$\begin{aligned} \Phi(t) =&\frac{1}{t} \Biggl\{ S(t)-S(0)+\sum _{i=1}^{2} \bigl(I_{i}(t)-I_{i}(0) \bigr)+\frac{\delta}{u+\delta}\bigl(V(t)-V(0)\bigr) \\ &{}-\sum_{i=1}^{2} \int_{0}^{t} \biggl[\sigma_{i}I_{i} \,dB_{i}(s)+ \int_{\mathbb{Z}}\gamma _{i}(u)I_{i}(s) \widetilde{N}(dt,du) \biggr] \\ &{}- \int_{0}^{t} \biggl[\sigma_{3}S \,dB_{3}(s)+ \int_{\mathbb{Z}}\gamma _{3}(u)S(s)\widetilde{N}(dt,du) \biggr] \\ &{}-\frac{\delta}{u+\delta} \int_{0}^{t} \biggl[\sigma_{4}V \,dB_{4}(s)+ \int_{\mathbb{Z}}\gamma_{4}(u)V(s)\widetilde{N}(dt,du) \biggr] \Biggr\} . \end{aligned}$$

Applying Lemmas 2.7 and 2.8, we obtain that

13 $$ \lim_{t\rightarrow+\infty}\Phi(t)=0\quad \mbox{a.s.} $$

Applying the generalized Itô’s formula in Lemma 2.5 to $\alpha_{1}\ln I_{1}(t)+I_{1}(t)$ yields

14 $$\begin{aligned} d\bigl[\alpha_{1}\ln I_{1}(t)+I_{1}(t) \bigr] =& \bigl[\beta_{1}S-(u+d_{1}+r_{1})I_{1}- \alpha _{1}(u+d_{1}+r_{1})-\alpha_{1}b_{1} \bigr]\,dt \\ &{}+(\alpha_{1}+I_{1})\sigma_{1} \,dB_{1}(t)+ \int_{\mathbb{Z}}\bigl[\alpha_{1}\ln\bigl(1+ \gamma_{1}(u)\bigr) \\ &{}+I_{1}\gamma_{1}(u)\bigr] \widetilde {N}(dt,du). \end{aligned}$$

Dividing both sides of equation (14) by *t*, integrating over the time interval 0 to *t* and taking the limit, one obtains that

15 $$\begin{aligned} \frac{\alpha_{1}\ln I_{1}(t)+I_{1}(t)}{t} =&\frac{\alpha_{1}\ln I_{1}(0)+I_{1}(0)}{t}+\beta_{1}\bigl\langle S(t)\bigr\rangle -(u+d_{1}+r_{1})\bigl\langle I_{1}(t)\bigr\rangle \\ &{}-\alpha_{1}(u+d_{1}+r_{1})- \alpha_{1}b_{1}+\frac{1}{t} \int_{0}^{t}\bigl(\alpha _{1}+I_{1}(s) \bigr)\sigma_{1}\,dB_{1}(s) \\ &{}+\frac{1}{t} \int_{0}^{t} \int_{\mathbb{Z}}\bigl[\alpha_{1}\ln\bigl(1+\gamma _{1}(u)\bigr)+I_{1}(s)\gamma_{1}(u)\bigr] \widetilde{N}(dt,du). \end{aligned}$$

Combining equations (12) and (15), one obtains

16 $$\begin{aligned} \frac{\alpha_{1}\ln I_{1}(t)}{t} =&\frac{\beta_{1}(u+\delta-uq)\Lambda }{u^{2}+u\delta+up}-\alpha_{1} (u+d_{1}+r_{1}+b_{1} )-\frac{\beta _{1}(u+\delta)(u+d_{2})}{u^{2}+u\delta+up}\bigl\langle I_{2}(t)\bigr\rangle \\ &{}- \biggl(\frac{\beta_{1}(u+\delta)(u+d_{1})}{u^{2}+u\delta +up}+(u+d_{1}+r_{1}) \biggr) \bigl\langle I_{1}(t)\bigr\rangle +\frac{\alpha\ln I_{1}(0)+I_{1}(0)}{t} \\ &{}-\frac{I_{1}(t)}{t}-\frac{\beta_{1}(u+\delta)}{u^{2}+u\delta+up}\Phi(t)+\frac {1}{t} \int_{0}^{t}\bigl(\alpha_{1}+I_{1}(s) \bigr)\sigma_{1}\,dB_{1}(s) \\ &{}+\frac{1}{t} \int_{0}^{t} \int_{\mathbb{Z}}\bigl[\alpha_{1}\ln\bigl(1+\gamma _{1}(u)\bigr)+I_{1}(s)\gamma_{1}(u)\bigr] \widetilde{N}(dt,du) \\ =&\frac{\beta_{1}(u+\delta-uq)\Lambda}{u^{2}+u\delta+up}-\alpha_{1} (u+d_{1}+r_{1}+b_{1} )-\frac{\beta_{1}(u+\delta)(u+d_{2})}{u^{2}+u\delta +up}\bigl\langle I_{2}(t)\bigr\rangle \\ &{}- \biggl(\frac{\beta_{1}(u+\delta)(u+d_{1})}{u^{2}+u\delta +up}+(u+d_{1}+r_{1}) \biggr) \bigl\langle I_{1}(t)\bigr\rangle +\Psi_{1}(t), \end{aligned}$$

where

$$\begin{aligned} \Psi_{1}(t) =&\frac{\alpha_{1}\ln I_{1}(0)+I_{1}(0)}{t}-\frac{I_{1}(t)}{t}- \frac {\beta_{1}(u+\delta)}{u^{2}+u\delta+up}\Phi(t) \\ &{}+\frac{1}{t} \int_{0}^{t}\bigl(\alpha _{1}+I_{1}(s) \bigr)\sigma_{1}\,dB_{1}(s) \\ &{}+\frac{1}{t} \int_{0}^{t} \int_{\mathbb{Z}}\bigl[\alpha_{1}\ln\bigl(1+\gamma _{1}(u)\bigr)+I_{1}(s)\gamma_{1}(u)\bigr] \widetilde{N}(dt,du). \end{aligned}$$

Similarly, applying the generalized Itô’s formula in Lemma 2.5 to $\alpha_{2}\ln I_{2}(t)+I_{2}(t)$ yields

17 $$\begin{aligned} \frac{\alpha_{2}\ln I_{2}(t)}{t} =&\frac{\beta_{2}(u+\delta-uq)\Lambda }{u^{2}+u\delta+up}-\alpha_{2} (u+d_{2}+r_{2}+b_{2} )-\frac{\beta _{2}(u+\delta)(u+d_{1})}{u^{2}+u\delta+up}\bigl\langle I_{1}(t)\bigr\rangle \\ &{}- \biggl(\frac{\beta_{2}(u+\delta)(u+d_{2})}{u^{2}+u\delta +up}+(u+d_{2}+r_{2}) \biggr)\bigl\langle I_{2}(t)\bigr\rangle +\Psi_{2}(t), \end{aligned}$$

where

$$\begin{aligned} \begin{aligned} \Psi_{2}(t)={}&\frac{\alpha_{2}\ln I_{2}(0)+I_{2}(0)}{t}-\frac{I_{2}(t)}{t}- \frac {\beta_{2}(u+\delta)}{u^{2}+u\delta+up}\Phi(t) \\ &{}+\frac{1}{t} \int_{0}^{t}\bigl(\alpha _{2}+I_{2}(s) \bigr)\sigma_{2}\,dB_{2}(s) \\ &{}+\frac{1}{t} \int_{0}^{t} \int_{\mathbb{Z}}\bigl[\alpha_{2}\ln\bigl(1+\gamma _{2}(u)\bigr)+I_{2}(s)\gamma_{2}(u)\bigr] \widetilde{N}(dt,du). \end{aligned} \end{aligned}$$

Applying Lemmas 2.7 and 2.8, we obtain that

18 $$ \lim_{t\rightarrow+\infty}\Psi_{i}(t)=0,\quad i=1,2 \mbox{ a.s.} $$

By taking the limit superior of both sides of equation (16) and equation (17), respectively, one has

$$\begin{aligned}& \limsup_{t\rightarrow\infty}\frac{\alpha_{1}\ln I_{1}(t)}{t}\leq\frac{\beta _{1}(u+\delta-uq)\Lambda}{u^{2}+u\delta+up}- \alpha_{1} (u+d_{1}+r_{1}+b_{1} )=R_{1}< 0, \\& \limsup_{t\rightarrow\infty}\frac{\alpha_{2}\ln I_{2}(t)}{t}\leq\frac{\beta _{2}(u+\delta-uq)\Lambda}{u^{2}+u\delta+up}- \alpha_{2} (u+d_{2}+r_{2}+b_{2} )=R_{2}< 0. \end{aligned}$$

That is to say,

19 $$ \lim_{t\rightarrow\infty}I_{i}(t)=0,\quad i=1,2 \mbox{ a.s.} $$

Applying (13) and (19) into equation (12), we obtain that

20 $$\begin{aligned} \lim_{t\rightarrow\infty}\bigl\langle S(t)\bigr\rangle =& \frac{u+\delta }{u^{2}+u\delta+up} \Biggl[\frac{(u+\delta-uq)\Lambda}{u+\delta}-\sum_{i=1}^{2} (u+d_{i} )\lim_{t\rightarrow\infty}\bigl\langle I_{i}(t) \bigr\rangle -\lim_{t\rightarrow\infty}\Phi(t) \Biggr] \\ =&\frac{(u+\delta-uq)\Lambda}{u^{2}+u\delta+up}. \end{aligned}$$

By equation (4), one has

21 $$\begin{aligned} d(S+I_{1}+I_{2}+V) =& \bigl[ \Lambda-uS-uV-(u+d_{1})I_{1}-(u+d_{2})I_{2} \bigr]\,dt \\ &{}+\sum_{i=1}^{2} \biggl[ \sigma_{i}I_{i}\,dB_{i}(t)+ \int_{\mathbb{Z}}\gamma _{i}(u)I_{i}\bigl(t^{-} \bigr)\widetilde{N}(dt,du) \biggr] \\ &{}+\sigma_{3}S\,dB_{3}(t)+ \int_{\mathbb{Z}}\gamma_{3}(u)S\bigl(t^{-}\bigr)\widetilde {N}(dt,du) \\ &{}+\sigma_{4}V\,dB_{4}(t)+ \int_{\mathbb{Z}}\gamma_{4}(u)V\bigl(t^{-}\bigr)\widetilde {N}(dt,du). \end{aligned}$$

Dividing both sides of equation (21) by *t*, integrating over the time interval $t=0$ to *t* and taking the limit, one obtains that

22 $$\begin{aligned} \lim_{t\rightarrow\infty}\bigl\langle V(t)\bigr\rangle =& \frac{\Lambda}{u}-\lim_{t\rightarrow\infty}\bigl\langle S(t)\bigr\rangle -\sum _{i=1}^{2}\frac{u+d_{i}}{u}\lim _{t\rightarrow\infty}\bigl\langle I_{i}(t)\bigr\rangle \\ &{}-\lim_{t\rightarrow\infty}\frac{S(t)-S(0)+\sum_{i=1}^{2}(I_{i}(t)-I_{i}(0))+V(t)-V(0)}{ut} \\ &{}+\frac{1}{u}\lim_{t\rightarrow\infty}\frac{1}{t} \int_{0}^{t} \Biggl\{ \sum _{i=1}^{2} \biggl[\sigma_{i}I_{i}(s) \,dB_{i}(s)+ \int_{\mathbb{Z}}\gamma _{1}(u)I_{1}\bigl(s^{-} \bigr)\widetilde{N}(ds,du) \biggr] \\ &{}+\sigma_{3}S(s)\,dB_{3}(s)+ \int_{\mathbb{Z}}\gamma_{3}(u)S\bigl(s^{-}\bigr)\widetilde {N}(ds,du) \\ &{}+\sigma_{4}V(s)\,dB_{4}(s)+ \int_{\mathbb{Z}}\gamma_{4}(u)V\bigl(s^{-}\bigr)\widetilde {N}(ds,du) \Biggr\} \,ds. \end{aligned}$$

Applying (19), (20), Lemmas 2.7 and 2.8, we have

$$ \lim_{t\rightarrow\infty}\bigl\langle V(t)\bigr\rangle = \frac{\Lambda}{u}-\frac {(u+\delta-uq)\Lambda}{u^{2}+u\delta+up}=\frac{(p+uq)\Lambda}{u^{2}+u\delta +up}. $$

This completes the proof. □

### Stochastic endemic dynamics

#### Theorem 2.2

*For any initial value* $(S(0),I_{1}(0),I_{2}(0),V(0))\in R_{+}^{4}$, *the solution* $(S(t),I_{1}(t), I_{2}(t),V(t))$ *of model* (4) *has the following property*:

(i) *If* $R_{1}>0$ *and* $R_{2}<0$, *then the epidemic disease* $I_{1}(t)$ *is persistent in mean and* $I_{2}(t)$ *goes extinct*, *i*.*e*. $\lim_{t\rightarrow\infty}\langle I_{1}(t)\rangle=\frac{R_{1}}{\Upsilon _{11}}>0$, $\lim_{t\rightarrow\infty}I_{2}(t)=0$ *a*.*s*. *Moreover*,
  $$\begin{aligned}& \lim_{t\rightarrow\infty}\bigl\langle S(t)\bigr\rangle = \frac{(u+\delta-uq)\Lambda }{u^{2}+u\delta+up}- \frac{(u+\delta)(u+d_{1})}{u^{2}+u\delta+up}\frac {R_{1}}{\Upsilon_{11}}\quad \textit{a.s.}, \\& \lim_{t\rightarrow\infty}\bigl\langle V(t)\bigr\rangle = \frac{(p+uq)\Lambda }{u^{2}+u\delta+up}- \frac{(u+d_{1})p}{u(u+\delta+p)}\frac{R_{1}}{\Upsilon _{11}} \quad \textit{a.s.} \end{aligned}$$
(ii) *If* $R_{1}<0$ *and* $R_{2}>0$, *then the epidemic disease* $I_{1}(t)$ *goes extinct and* $I_{2}(t)$ *is persistent in mean*, *i*.*e*. $\lim_{t\rightarrow\infty}\langle I_{1}(t)\rangle=0$, $\lim_{t\rightarrow \infty}I_{2}(t)=\frac{R_{2}}{\Upsilon_{21}}>0$ *a*.*s*. *Moreover*,
  $$\begin{aligned}& \lim_{t\rightarrow\infty}\bigl\langle S(t)\bigr\rangle = \frac{(u+\delta-uq)\Lambda }{u^{2}+u\delta+up}- \frac{(u+\delta)(u+d_{2})}{u^{2}+u\delta+up}\frac {R_{2}}{\Upsilon_{21}}\quad \textit{a.s.}, \\& \lim_{t\rightarrow\infty}\bigl\langle V(t)\bigr\rangle = \frac{(p+uq)\Lambda }{u^{2}+u\delta+up}- \frac{(u+d_{2})p}{u(u+\delta+p)}\frac{R_{2}}{\Upsilon _{21}}\quad \textit{a.s.} \end{aligned}$$

#### Proof

Case (i): From equation (16) we have

23 $$\begin{aligned} \frac{\alpha_{1}\ln I_{1}(t)}{t} =&\frac{\beta_{1}(u+\delta-uq)\Lambda }{u^{2}+u\delta+up}-\alpha_{1} (u+d_{1}+r_{1}+b_{1} ) \\ &{}- \biggl[ \frac{\beta _{1}(u+\delta)(u+d_{1})}{u^{2}+u\delta+up}+(u+d_{1}+r_{1}) \biggr]\bigl\langle I_{1}(t)\bigr\rangle \\ &{}-\frac{\beta_{1}(u+\delta)(u+d_{2})}{u^{2}+u\delta+up}\bigl\langle I_{2}(t)\bigr\rangle + \Psi_{1}(t) \\ =&R_{1}-\Upsilon_{11}\bigl\langle I_{1}(t)\bigr\rangle -\Upsilon_{12}\bigl\langle I_{2}(t)\bigr\rangle + \Psi_{1}(t), \end{aligned}$$

where

$$\Upsilon_{11}=\frac{\beta_{1}(u+\delta)(u+d_{1})}{u^{2}+u\delta +up}+(u+d_{1}+r_{1}), \qquad \Upsilon_{12}=\frac{\beta_{1}(u+\delta )(u+d_{2})}{u^{2}+u\delta+up}. $$

From Theorem 2.1, when $R_{2}<0$ one has

24 $$ \lim_{t\rightarrow\infty}I_{2}(t)=0 \quad \mbox{a.s.} $$

Therefore, there exists an arbitrarily small constant $\varepsilon>0$ such that when *t* is large enough, we have $I_{2}(t)<\varepsilon$. Applying this into equation (23) leads to

$$ R_{1}-\Upsilon_{11}\bigl\langle I_{1}(t)\bigr\rangle +\Psi_{1}(t)\geq \frac{\alpha_{1}\ln I_{1}(t)}{t}\geq R_{1}- \Upsilon_{11}\bigl\langle I_{1}(t)\bigr\rangle - \Upsilon_{12}\varepsilon+\Psi_{1}(t). $$

Applying Lemma 2.6 and the arbitrariness of *ε*, we obtain

25 $$ \lim_{t\rightarrow\infty}\bigl\langle I_{1}(t)\bigr\rangle =\frac{R_{1}}{\Upsilon _{11}}\quad \mbox{a.s.} $$

Applying (13), (24) and (25) into equation (12), we obtain that

26 $$\begin{aligned} \lim_{t\rightarrow\infty}\bigl\langle S(t)\bigr\rangle =& \frac{u+\delta }{u^{2}+u\delta+up} \Biggl[\frac{(u+\delta-uq)\Lambda}{u+\delta}-\sum_{i=1}^{2} (u+d_{i} )\lim_{t\rightarrow\infty}\bigl\langle I_{i}(t) \bigr\rangle -\lim_{t\rightarrow\infty}\Phi(t) \Biggr] \\ =&\frac{(u+\delta-uq)\Lambda}{u^{2}+u\delta+up}-\frac{(u+\delta )(u+d_{1})}{u^{2}+u\delta+up}\frac{R_{1}}{\Upsilon_{11}}. \end{aligned}$$

Applying (24), (25), (26), Lemmas 2.7 and 2.8 into equation (22), we have

$$\begin{aligned} \lim_{t\rightarrow\infty}\bigl\langle V(t)\bigr\rangle =& \frac{\Lambda}{u}-\frac {(u+\delta-uq)\Lambda}{u^{2}+u\delta+up}+\frac{(u+\delta )(u+d_{1})}{u^{2}+u\delta+up}\frac{R_{1}}{\Upsilon_{11}}- \frac{u+d_{1}}{u}\frac {R_{1}}{\Upsilon_{11}} \\ =&\frac{(p+uq)\Lambda}{u^{2}+u\delta+up}-\frac{(u+d_{1})p}{u(u+\delta +p)}\frac{R_{1}}{\Upsilon_{11}}. \end{aligned}$$

Case (ii): From equation (17) we have

27 $$\begin{aligned} \frac{\alpha_{2}\ln I_{2}(t)}{t} =&\frac{\beta_{2}(u+\delta-uq)\Lambda }{u^{2}+u\delta+up}-\alpha_{2} (u+d_{2}+r_{2}+b_{2} )-\frac{\beta _{2}(u+\delta)(u+d_{1})}{u^{2}+u\delta+up}\bigl\langle I_{1}(t)\bigr\rangle \\ &{}- \biggl[\frac{\beta_{2}(u+\delta)(u+d_{2})}{u^{2}+u\delta +up}+(u+d_{2}+r_{2}) \biggr]\bigl\langle I_{2}(t)\bigr\rangle +\Psi_{2}(t) \\ =&R_{2}-\Upsilon_{21}\bigl\langle I_{1}(t)\bigr\rangle -\Upsilon_{22}\bigl\langle I_{2}(t)\bigr\rangle + \Psi_{2}(t), \end{aligned}$$

where

$$\Upsilon_{21}=\frac{\beta_{2}(u+\delta)(u+d_{1})}{u^{2}+u\delta+up},\qquad \Upsilon _{22}= \frac{\beta_{2}(u+\delta)(u+d_{2})}{u^{2}+u\delta+up}+(u+d_{2}+r_{2}). $$

From Theorem 2.1, when $R_{1}<0$ one has

28 $$ \lim_{t\rightarrow\infty}I_{1}(t)=0\quad \mbox{a.s.} $$

Therefore, there exists an arbitrarily small constant $\varepsilon>0$ such that when *t* is large enough, we have $I_{1}(t)<\varepsilon$. Applying this into equation (23) leads to

$$ R_{2}-\Upsilon_{21}\bigl\langle I_{2}(t)\bigr\rangle +\Psi_{2}(t)\geq \frac{\alpha_{2}\ln I_{2}(t)}{t}\geq R_{2}- \Upsilon_{21}\bigl\langle I_{2}(t)\bigr\rangle - \Upsilon_{22}\varepsilon+\Psi_{2}(t). $$

Applying Lemma 2.6 and the arbitrariness of *ε*, we obtain

29 $$ \lim_{t\rightarrow\infty}\bigl\langle I_{2}(t)\bigr\rangle =\frac{R_{2}}{\Upsilon _{21}} \quad \mbox{a.s.} $$

Applying equations (13), (28), (29) into equation (12), we obtain that

30 $$\begin{aligned} \begin{aligned}[b] \lim_{t\rightarrow\infty}\bigl\langle S(t)\bigr\rangle &= \frac{u+\delta }{u^{2}+u\delta+up} \Biggl[\frac{(u+\delta-uq)\Lambda}{u+\delta}-\sum_{i=1}^{2} (u+d_{i} )\lim_{t\rightarrow\infty}\bigl\langle I_{i}(t) \bigr\rangle -\lim_{t\rightarrow\infty}\Phi(t) \Biggr] \\ &=\frac{(u+\delta-uq)\Lambda}{u^{2}+u\delta+up}-\frac{(u+\delta )(u+d_{2})}{u^{2}+u\delta+up}\frac{R_{2}}{\Upsilon_{21}}. \end{aligned} \end{aligned}$$

Applying (28), (29), (30), Lemmas 2.7 and 2.8 into equation (22), we have

$$\begin{aligned} \lim_{t\rightarrow\infty}\bigl\langle V(t)\bigr\rangle =& \frac{\Lambda}{u}-\frac {(u+\delta-uq)\Lambda}{u^{2}+u\delta+up}+\frac{(u+\delta )(u+d_{2})}{u^{2}+u\delta+up}\frac{R_{2}}{\Upsilon_{21}}- \frac{u+d_{1}}{u}\frac {R_{2}}{\Upsilon_{21}} \\ =&\frac{(p+uq)\Lambda}{u^{2}+u\delta+up}-\frac{(u+d_{2})p}{u(u+\delta +p)}\frac{R_{2}}{\Upsilon_{21}}. \end{aligned}$$

This completes the proof. □

#### Theorem 2.3

*Suppose that conditions* $R_{1}>0$ *and* $R_{2}>0$ *hold*. *Let* $(S(t),I_{1}(t),I_{2}(t),V(t))$ *be the solution of model* (4) *with the initial value* $(S(0),I_{1}(0),I_{2}(0),V(0))\in R_{+}^{4}$.

(i) *If* $\Upsilon_{11}R_{2}<\Upsilon_{21}R_{1}$, *then the epidemic disease* $I_{1}(t)$ *is persistent in mean and* $I_{2}(t)$ *goes extinct*, *i*.*e*. $\lim_{t\rightarrow\infty}\langle I_{1}(t)\rangle=\frac {R_{1}}{\Upsilon_{11}}>0$, $\lim_{t\rightarrow\infty}I_{2}(t)=0$ *a*.*s*. *Moreover*,
  $$\begin{aligned}& \lim_{t\rightarrow\infty}\bigl\langle S(t)\bigr\rangle = \frac{(u+\delta-uq)\Lambda }{u^{2}+u\delta+up}- \frac{(u+\delta)(u+d_{1})}{u^{2}+u\delta+up}\frac {R_{1}}{\Upsilon_{11}} \quad \textit{a.s.}, \\& \lim_{t\rightarrow\infty}\bigl\langle V(t)\bigr\rangle = \frac{(p+uq)\Lambda }{u^{2}+u\delta+up}- \frac{(u+d_{1})p}{u(u+\delta+p)}\frac{R_{1}}{\Upsilon _{11}}\quad \textit{a.s.} \end{aligned}$$
(ii) *If* $\Upsilon_{22}R_{1}<\Upsilon_{12}R_{2}$, *then the epidemic disease* $I_{1}(t)$ *goes extinct and* $I_{2}(t)$ *is persistent in mean*, *i*.*e*. $\lim_{t\rightarrow\infty}\langle I_{1}(t)\rangle=0$, $\lim_{t\rightarrow\infty}I_{2}(t)=\frac{R_{2}}{\Upsilon_{21}}>0$ *a*.*s*. *Moreover*,
  $$\begin{aligned}& \lim_{t\rightarrow\infty}\bigl\langle S(t)\bigr\rangle =\frac{(u+\delta-uq)\Lambda }{u^{2}+u\delta+up}- \frac{(u+\delta)(u+d_{2})}{u^{2}+u\delta+up}\frac {R_{2}}{\Upsilon_{21}} \quad \textit{a.s.}, \\& \lim_{t\rightarrow\infty}\bigl\langle V(t)\bigr\rangle =\frac{(p+uq)\Lambda }{u^{2}+u\delta+up}- \frac{(u+d_{2})p}{u(u+\delta+p)}\frac{R_{2}}{\Upsilon _{21}}\quad \textit{a.s.} \end{aligned}$$
(iii) *If* $\Upsilon_{11}R_{2}>\Upsilon_{21}R_{1}$, $\Upsilon _{22}R_{1}>\Upsilon_{12}R_{2}$, *then the epidemic diseases* $I_{1}$ *and* $I_{2}$ *are persistent in mean*. *Moreover*,
  $$\begin{aligned}& \lim_{t\rightarrow\infty}\bigl\langle I_{1}(t)\bigr\rangle = \frac{\Upsilon _{22}R_{1}-\Upsilon_{12}R_{2}}{\Upsilon_{11}\Upsilon_{22}-\Upsilon _{12}\Upsilon_{21}}, \qquad \lim_{t\rightarrow\infty}\bigl\langle I_{2}(t)\bigr\rangle =\frac{\Upsilon_{11}R_{2}-\Upsilon_{21}R_{1}}{\Upsilon_{11}\Upsilon _{22}-\Upsilon_{12}\Upsilon_{21}}\quad \textit{a.s.}, \\& \lim_{t\rightarrow\infty}\bigl\langle S(t)\bigr\rangle = \frac{(u+\delta-uq)\Lambda }{u^{2}+u\delta+up}- \frac{(u+\delta)(u+d_{1})}{u^{2}+u\delta+up}\frac{\Upsilon _{22}R_{1}-\Upsilon_{12}R_{2}}{\Upsilon_{11}\Upsilon_{22}-\Upsilon _{12}\Upsilon_{21}} \\& \hphantom{\lim_{t\rightarrow\infty}\bigl\langle S(t)\bigr\rangle ={}}{}-\frac{(u+\delta)(u+d_{2})}{u^{2}+u\delta+up}\frac{\Upsilon _{11}R_{2}-\Upsilon_{21}R_{1}}{\Upsilon_{11}\Upsilon_{22}-\Upsilon _{12}\Upsilon_{21}}\quad \textit{a.s.}, \\& \lim_{t\rightarrow\infty}\bigl\langle V(t)\bigr\rangle = \frac{(p+uq)\Lambda }{u^{2}+u\delta+up}- \frac{(u+d_{1})p}{u(u+\delta+p)}\frac{\Upsilon _{22}R_{1}-\Upsilon_{12}R_{2}}{\Upsilon_{11}\Upsilon_{22}-\Upsilon _{12}\Upsilon_{21}} \\& \hphantom{\lim_{t\rightarrow\infty}\bigl\langle V(t)\bigr\rangle ={}}{}-\frac{(u+d_{2})}{u(u+\delta+p)}\frac{\Upsilon_{11}R_{2}-\Upsilon _{21}R_{1}}{\Upsilon_{11}\Upsilon_{22}-\Upsilon_{12}\Upsilon_{21}}\quad \textit{a.s.} \end{aligned}$$

#### Proof

Case (i): Note that

$$\limsup_{t\rightarrow+\infty}\frac{\ln I_{1}(t)}{t}\leq0, $$

there exists an arbitrarily small constant $\varepsilon>0$ such that when *t* is large enough, we have

$$\frac{\ln I_{1}(t)}{t}< \varepsilon. $$

From equation (23) and equation (27), when *t* is large enough, one has

31 $$\begin{aligned} \frac{\Upsilon_{11}\alpha_{2}\ln I_{2}(t)}{t} =&\Upsilon_{11}R_{2}- \Upsilon_{21}R_{1}-(\Upsilon_{11} \Upsilon_{22}-\Upsilon _{12}\Upsilon_{21})\bigl\langle I_{2}(t)\bigr\rangle +\Upsilon_{21} \alpha_{1}\frac{\ln I_{1}(t)}{t} \\ &{}+\Upsilon_{11}\Psi_{2}(t)-\Upsilon_{21} \Psi_{1}(t) \\ \leq&\Upsilon_{11}R_{2}-\Upsilon_{21}R_{1}-( \Upsilon_{11}\Upsilon _{22}-\Upsilon_{12} \Upsilon_{21})\bigl\langle I_{2}(t)\bigr\rangle +\Upsilon _{21}\alpha_{1}\varepsilon \\ &{}+\Upsilon_{11}\Psi_{2}(t)-\Upsilon_{21} \Psi_{1}(t). \end{aligned}$$

Since $\Upsilon_{11}R_{2}<\Upsilon_{21}R_{1}$ and $\Upsilon_{11}\Upsilon _{22}>\Upsilon_{12}\Upsilon_{21}$, taking the limit superior of both sides of equation (31), applying equation (18) and the arbitrariness of *ε*, we have

$$ \limsup_{t\rightarrow+\infty}\frac{\ln I_{2}(t)}{t}\leq \frac{\Upsilon _{11}R_{2}-\Upsilon_{21}R_{1}}{\Upsilon_{11}\alpha_{2}}< 0. $$

That is to say,

$$ \lim_{t\rightarrow\infty}I_{2}(t)=0\quad \mbox{a.s.} $$

By using the method of Case (ii) in Theorem 2.2, one obtains the persistence in mean of $I_{1}(t)$, $S(t)$ and $V(t)$, and hence is omitted.

Case (ii): The proof of Case (ii) is similar to the proof of Case (i) in this subsection and hence is omitted.

Case (iii): Since $\Upsilon_{11}R_{2}>\Upsilon_{21}R_{1}$ and $\Upsilon_{11}\Upsilon_{22}>\Upsilon_{12}\Upsilon_{21}$, using Lemma 2.6 and the arbitrariness of *ε* for equation (31), one obtains that

32 $$ \limsup_{t\rightarrow+\infty}\bigl\langle I_{2}(t) \bigr\rangle \leq\frac{\Upsilon _{11}R_{2}-\Upsilon_{21}R_{1}}{\Upsilon_{11}\Upsilon_{22}-\Upsilon _{12}\Upsilon_{21}} \quad \mbox{a.s.} $$

Similarly, when $\Upsilon_{22}R_{1}>\Upsilon_{12}R_{2}$, we have

33 $$ \limsup_{t\rightarrow+\infty}\bigl\langle I_{1}(t) \bigr\rangle \leq\frac{\Upsilon _{22}R_{1}-\Upsilon_{12}R_{2}}{\Upsilon_{11}\Upsilon_{22}-\Upsilon _{12}\Upsilon_{21}} \quad \mbox{a.s.} $$

From equation (32), there exists an arbitrarily small constant $\varepsilon>0$ such that when *t* is large enough, we have

34 $$ \bigl\langle I_{2}(t)\bigr\rangle \leq \frac{\Upsilon_{11}R_{2}-\Upsilon _{21}R_{1}}{\Upsilon_{11}\Upsilon_{22}-\Upsilon_{12}\Upsilon _{21}}+\varepsilon. $$

Applying equation (23) into equation (34), one obtains that

$$\begin{aligned} \frac{\alpha_{1}\ln I_{1}(t)}{t} =&R_{1}-\Upsilon_{11}\bigl\langle I_{1}(t)\bigr\rangle -\Upsilon_{12}\bigl\langle I_{2}(t)\bigr\rangle +\Psi_{1}(t) \\ \geq&R_{1}-\Upsilon_{11}\bigl\langle I_{1}(t) \bigr\rangle -\Upsilon_{12}\varepsilon -\Upsilon_{12} \frac{\Upsilon_{11}R_{2}-\Upsilon_{21}R_{1}}{\Upsilon _{11}\Upsilon_{22}-\Upsilon_{12}\Upsilon_{21}}+\Psi_{1}(t). \end{aligned}$$

By using Lemma 2.6 and the arbitrariness of *ε*, we obtain that

35 $$ \liminf_{t\rightarrow+\infty}\bigl\langle I_{1}(t) \bigr\rangle \geq\frac{\Upsilon _{22}R_{1}-\Upsilon_{12}R_{2}}{\Upsilon_{11}\Upsilon_{22}-\Upsilon _{12}\Upsilon_{21}}\quad \mbox{a.s.} $$

Similarly, one obtains

36 $$ \liminf_{t\rightarrow+\infty}\bigl\langle I_{2}(t) \bigr\rangle \geq\frac{\Upsilon _{11}R_{2}-\Upsilon_{21}R_{1}}{\Upsilon_{11}\Upsilon_{22}-\Upsilon _{12}\Upsilon_{21}} \quad \mbox{a.s.} $$

Applying equations (32), (33), (35) and (36) leads to

37 $$ \lim_{t\rightarrow+\infty}\bigl\langle I_{1}(t)\bigr\rangle =\frac{\Upsilon _{22}R_{1}-\Upsilon_{12}R_{2}}{\Upsilon_{11}\Upsilon_{22}-\Upsilon _{12}\Upsilon_{21}},\qquad \lim_{t\rightarrow+\infty}\bigl\langle I_{2}(t)\bigr\rangle =\frac{\Upsilon_{11}R_{2}-\Upsilon_{21}R_{1}}{\Upsilon_{11}\Upsilon _{22}-\Upsilon_{12}\Upsilon_{21}} \quad \mbox{a.s.} $$

Applying (13) and (37) into equation (12), we obtain that

38 $$\begin{aligned} \lim_{t\rightarrow\infty}\bigl\langle S(t)\bigr\rangle =& \frac{u+\delta }{u^{2}+u\delta+up} \Biggl[\frac{(u+\delta-uq)\Lambda}{u+\delta}-\sum_{i=1}^{2} (u+d_{i} )\lim_{t\rightarrow\infty}\bigl\langle I_{i}(t) \bigr\rangle -\lim_{t\rightarrow\infty}\Phi(t) \Biggr] \\ =&\frac{(u+\delta-uq)\Lambda}{u^{2}+u\delta+up}-\frac{(u+\delta )(u+d_{1})}{u^{2}+u\delta+up}\frac{\Upsilon_{22}R_{1}-\Upsilon _{12}R_{2}}{\Upsilon_{11}\Upsilon_{22}-\Upsilon_{12}\Upsilon_{21}} \\ &{}-\frac{(u+\delta)(u+d_{2})}{u^{2}+u\delta+up}\frac{\Upsilon _{11}R_{2}-\Upsilon_{21}R_{1}}{\Upsilon_{11}\Upsilon_{22}-\Upsilon _{12}\Upsilon_{21}}. \end{aligned}$$

Applying (37), (38), Lemmas 2.7 and 2.8 into equation (22), we have

$$\begin{aligned} \lim_{t\rightarrow\infty}\bigl\langle V(t)\bigr\rangle =&\frac{\Lambda}{u}- \frac {(u+\delta-uq)\Lambda}{u^{2}+u\delta+up}+\frac{(u+\delta )(u+d_{1})}{u^{2}+u\delta+up}\frac{\Upsilon_{22}R_{1}-\Upsilon _{12}R_{2}}{\Upsilon_{11}\Upsilon_{22}-\Upsilon_{12}\Upsilon_{21}} \\ &{}-\frac {u+d_{1}}{u} \frac{\Upsilon_{22}R_{1}-\Upsilon_{12}R_{2}}{\Upsilon_{11}\Upsilon _{22}-\Upsilon_{12}\Upsilon_{21}}+\frac{(u+\delta)(u+d_{2})}{u^{2}+u\delta+up}\frac{\Upsilon _{11}R_{2}-\Upsilon_{21}R_{1}}{\Upsilon_{11}\Upsilon_{22}-\Upsilon _{12}\Upsilon_{21}} \\ &{}-\frac{u+d_{2}}{u} \frac{\Upsilon_{11}R_{2}-\Upsilon _{21}R_{1}}{\Upsilon_{11}\Upsilon_{22}-\Upsilon_{12}\Upsilon_{21}} \\ =&\frac{(p+uq)\Lambda}{u^{2}+u\delta+up}-\frac{(u+d_{1})p}{u(u+\delta +p)}\frac{\Upsilon_{22}R_{1}-\Upsilon_{12}R_{2}}{\Upsilon_{11}\Upsilon _{22}-\Upsilon_{12}\Upsilon_{21}} \\ &{}- \frac{(u+d_{2})p}{u(u+\delta+p)}\frac {\Upsilon_{11}R_{2}-\Upsilon_{21}R_{1}}{\Upsilon_{11}\Upsilon_{22}-\Upsilon _{12}\Upsilon_{21}}. \end{aligned}$$

This completes the proof. □

## Conclusions and numerical simulations

In this paper, we propose a novel stochastic epidemic system with double epidemic diseases under vaccination. By using stochastic differential equation theory, we study the persistence in mean and extinction of the two diseases. Compared with the existing work in Refs. [39] and [40], the model constructed in this paper also considers the efficiency of vaccination. When all the coefficients related to the vaccination are 0, system (4) is similar to systems (2) and (3) in Refs. [39] and [40], in addition, our conclusion is consistent with them. That is to say, systems (2) and (3) in Refs. [39] and [40] are a special case of our system (4). The theoretical results of this article can be used as a reference for the control of infectious diseases.

To sum up, we have the following conclusions:

I.: Stochastic disease-free dynamics
  When $R_{1}<0$ and $R_{2}<0$ hold, we have
  $$\begin{aligned}& \lim_{t\rightarrow\infty}I_{i}(t)=0,\quad i=1,2,\qquad \lim _{t\rightarrow\infty }\bigl\langle S(t)\bigr\rangle =\frac{(u+\delta-uq)\Lambda}{u^{2}+u\delta+up}, \\& \lim_{t\rightarrow\infty}\bigl\langle V(t)\bigr\rangle =\frac{(p+uq)\Lambda }{u^{2}+u\delta+up}. \end{aligned}$$
  That is to say, the two epidemic diseases go to extinct almost surely.
II.: Stochastic endemic dynamics
  (i) If one of the following conditions holds:
$R_{1}>0$, $R_{2}<0$,
$R_{1}, R_{2}>0$, $\Upsilon_{11}R_{2}<\Upsilon_{21}R_{1}$,
    then we have
    $$\begin{aligned}& \lim_{t\rightarrow\infty}\bigl\langle I_{1}(t)\bigr\rangle = \frac{R_{1}}{\Upsilon _{11}}>0,\qquad \lim_{t\rightarrow\infty}I_{2}(t)=0\quad \mbox{a.s.}, \\& \lim_{t\rightarrow\infty}\bigl\langle S(t)\bigr\rangle = \frac{(u+\delta-uq)\Lambda }{u^{2}+u\delta+up}- \frac{(u+\delta)(u+d_{1})}{u^{2}+u\delta+up}\frac {R_{1}}{\Upsilon_{11}} \quad \mbox{a.s.}, \\& \lim_{t\rightarrow\infty}\bigl\langle V(t)\bigr\rangle = \frac{(p+uq)\Lambda }{u^{2}+u\delta+up}- \frac{(u+d_{1})p}{u(u+\delta+p)}\frac{R_{1}}{\Upsilon _{11}}\quad \mbox{a.s.} \end{aligned}$$
    That is to say, the epidemic disease $I_{1}(t)$ is persistent in mean and $I_{2}(t)$ is extinct.
  (ii) If one of the following conditions hold:
$R_{1}<0$, $R_{2}>0$,
$R_{1}, R_{2}>0$, $\Upsilon_{22}R_{1}<\Upsilon_{12}R_{2}$,
    then we have
    $$\begin{aligned}& \lim_{t\rightarrow\infty}\bigl\langle I_{1}(t)\bigr\rangle = 0, \qquad \lim_{t\rightarrow \infty}I_{2}(t)=\frac{R_{2}}{\Upsilon_{21}}>0\quad \mbox{a.s.}, \\& \lim_{t\rightarrow\infty}\bigl\langle S(t)\bigr\rangle = \frac{(u+\delta-uq)\Lambda }{u^{2}+u\delta+up}- \frac{(u+\delta)(u+d_{2})}{u^{2}+u\delta+up}\frac {R_{2}}{\Upsilon_{21}}\quad \mbox{a.s.}, \\& \lim_{t\rightarrow\infty}\bigl\langle V(t)\bigr\rangle = \frac{(p+uq)\Lambda }{u^{2}+u\delta+up}- \frac{(u+d_{2})p}{u(u+\delta+p)}\frac{R_{2}}{\Upsilon _{21}} \quad \mbox{a.s.} \end{aligned}$$
    That is to say, the epidemic disease $I_{1}(t)$ is extinct and $I_{2}(t)$ is persistent in mean.
  (iii) If $\Upsilon_{11}R_{2}>\Upsilon_{21}R_{1}$, $\Upsilon _{22}R_{1}>\Upsilon_{12}R_{2}$ hold, then we have
    $$\begin{aligned}& \lim_{t\rightarrow\infty}\bigl\langle I_{1}(t)\bigr\rangle = \frac{\Upsilon _{22}R_{1}-\Upsilon_{12}R_{2}}{\Upsilon_{11}\Upsilon_{22}-\Upsilon _{12}\Upsilon_{21}}, \qquad \lim_{t\rightarrow\infty}\bigl\langle I_{2}(t)\bigr\rangle =\frac{\Upsilon_{11}R_{2}-\Upsilon_{21}R_{1}}{\Upsilon_{11}\Upsilon _{22}-\Upsilon_{12}\Upsilon_{21}} \quad \mbox{a.s.}, \\& \lim_{t\rightarrow\infty}\bigl\langle S(t)\bigr\rangle = \frac{(u+\delta-uq)\Lambda }{u^{2}+u\delta+up}- \frac{(u+\delta)(u+d_{1})}{u^{2}+u\delta+up}\frac{\Upsilon _{22}R_{1}-\Upsilon_{12}R_{2}}{\Upsilon_{11}\Upsilon_{22}-\Upsilon _{12}\Upsilon_{21}} \\& \hphantom{\lim_{t\rightarrow\infty}\bigl\langle S(t)\bigr\rangle ={}}{}-\frac{(u+\delta)(u+d_{2})}{u^{2}+u\delta+up}\frac{\Upsilon _{11}R_{2}-\Upsilon_{21}R_{1}}{\Upsilon_{11}\Upsilon_{22}-\Upsilon _{12}\Upsilon_{21}}\quad \mbox{a.s.}, \\& \lim_{t\rightarrow\infty}\bigl\langle V(t)\bigr\rangle = \frac{(p+uq)\Lambda }{u^{2}+u\delta+up}- \frac{(u+d_{1})p}{u(u+\delta+p)}\frac{\Upsilon _{22}R_{1}-\Upsilon_{12}R_{2}}{\Upsilon_{11}\Upsilon_{22}-\Upsilon _{12}\Upsilon_{21}} \\& \hphantom{\lim_{t\rightarrow\infty}\bigl\langle V(t)\bigr\rangle ={}}{}-\frac{(u+d_{2})}{u(u+\delta+p)}\frac{\Upsilon_{11}R_{2}-\Upsilon _{21}R_{1}}{\Upsilon_{11}\Upsilon_{22}-\Upsilon_{12}\Upsilon_{21}} \quad \mbox{a.s.} \end{aligned}$$
    That is to say, the epidemic diseases $I_{1}$ and $I_{2}$ are persistent in mean.

In [39, 41], Meng and Chang et al. obtained the lower boundedness of the persistence in mean for $I_{1}$ and $I_{2}$ as follows:

$$\liminf_{t\rightarrow+\infty}\bigl\langle I_{1}(t) + I_{2}(t)\bigr\rangle \geq m^{*}, $$

where $m^{*}$ is a positive constant. However, this paper proves that $I_{1}$ and $I_{2}$ have their own limit, that is,

$$\lim_{t\rightarrow+\infty}\bigl\langle I_{1}(t)\bigr\rangle = m_{1}^{*}, \qquad \lim_{t\rightarrow+\infty}\bigl\langle I_{2}(t)\bigr\rangle = m_{2}^{*}, $$

where $m_{1}^{*}=\frac{\Upsilon_{22}R_{1}-\Upsilon_{12}R_{2}}{\Upsilon _{11}\Upsilon_{22}-\Upsilon_{12}\Upsilon_{21}}$ and $m_{2}^{*}=\frac {\Upsilon_{11}R_{2}-\Upsilon_{21}R_{1}}{\Upsilon_{11}\Upsilon_{22}-\Upsilon _{12}\Upsilon_{21}}$. Thus this paper contains and significantly improves the results for persistence in mean in [39, 41]. The developed theoretical methods can be used to investigate the high-dimensional nonlinear stochastic differential systems.

To numerically illustrate our results, we employ a numerical method from [52] with ©Matlab2013b to the following discrete equations:

$$ \textstyle\begin{cases} S_{n+1}= S_{n}+ [(1-q)\Lambda-(u+p)S_{n}-\frac{\beta_{1}S_{n}I_{1,n}}{\alpha _{1}+I_{1,n}}-\frac{\beta_{2}S_{n}I_{2,n}}{\alpha _{2}+I_{2,n}}+r_{1}I_{1,n}+r_{2}I_{2,n}+\delta V_{n} ]\Delta t \\ \hphantom{S_{n+1}={}}{}+\sigma_{3}S_{n}\Delta W_{1k}+S_{n}\gamma_{3}\Delta\Gamma_{3k}, \\ I_{1,n+1}= I_{1,n}+ [\frac{\beta_{1}S_{n}I_{1,n}}{\alpha _{1}+I_{1,n}}-(u+d_{1}+r_{1})I_{1,n} ]\Delta t+\sigma_{1}I_{1,n}\Delta W_{1k}+I_{1,n}\gamma_{1}\Delta\Gamma_{1k}, \\ I_{2,n+1}= I_{2,n}+ [\frac{\beta_{2}S_{n}I_{2,n}}{\alpha _{2}+I_{2,n}}-(u+d_{2}+r_{2})I_{2,n} ]\Delta t+\sigma_{2}I_{2,n}\Delta W_{2k}+I_{2,n}\gamma_{2}\Delta\Gamma_{2k}, \\ V_{n+1}= V_{n}+ [q\Lambda+pS_{n}-(u+\delta)V_{n} ]\Delta t+\sigma _{4}V_{n}\Delta W_{4k}+V_{n}\gamma_{4}\Delta\Gamma_{4k}, \end{cases} $$

where $\Delta t=0.01$, $\Delta W_{ik}\triangleq W(t_{k+1})-W(t_{k})$ ($i=1,2,3,4$) obeys the Gaussian distribution $N(0,\Delta t)$, $\Delta \Gamma_{ik}\triangleq\Gamma(t_{k+1})-\Gamma(t_{k})$ obeys the Poisson distribution with intensity *λ*.

To this end, we set $\Lambda=1$, $q=0.1$, $u=0.2$, $p=0.2$, $\beta_{1}=0.24$, $\beta _{2}=0.27$, $\alpha_{1}=1$, $\alpha_{2}=1$, $r_{1}=0.2$, $r_{2}=0.1$, $\delta=0.2$, $d_{1}=0.2$, $d_{2}=0.4$.

**Figure 1:**
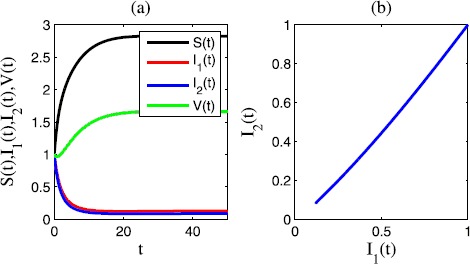
**Time sequence diagram and phase diagram of model** (**4**) **without stochastic effects.**

Figure 1(a) is the time sequence diagram of system (4) with $\sigma_{i}=\gamma_{i}=0$, $i=1,2,3,4$; Figure 1(b) is the corresponding phase diagram of $I_{1}(t)$ and $I_{2}(t)$. In this case, the two epidemic diseases are persistent.

**Figure 2:**
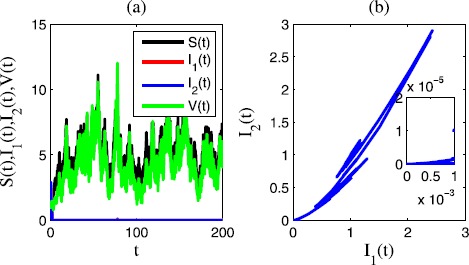
**Time sequence diagram and phase diagram of model** (**4**) **for extinction of two epidemic diseases.**

In Figure 2, we choose $\sigma_{1}=0.6$, $\sigma_{2}=0.8$, $\sigma_{3}=0.1$, $\sigma _{4}=0.1$, $\gamma_{1}=0.2$, $\gamma_{2}=0.3$, $\gamma_{3}=0.1$, $\gamma_{4}=0.2$. In this case, $R_{1}=-0.0377<0$, $R_{2}=-0.2026<0$. We see that in the time sequence diagram Figure 2(a) and the corresponding phase diagram Figure 2(b), the two epidemic diseases are extinct.

**Figure 3:**
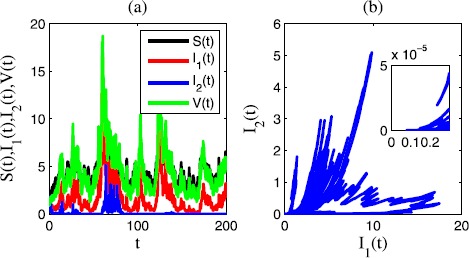
**Time sequence diagram and phase diagram of model** (**4**) **for extinctions of disease 2 and persistence of disease 1.**

In Figure 3, we choose $\sigma_{1}=0.2$, $\sigma_{2}=0.6$, $\sigma_{3}=0.1$, $\sigma _{4}=0.1$, $\gamma_{1}=0.3$, $\gamma_{2}=0.3$, $\gamma_{3}=0.1$, $\gamma_{4}=0.2$. In this case, $R_{1}=0.1024>0$, $R_{2}=-0.0626<0$. We see that in the time sequence diagram Figure 3(a) and the corresponding phase diagram Figure 3(b), the epidemic disease $I_{1}(t)$ is persistent in mean and $I_{2}(t)$ is extinct.

**Figure 4:**
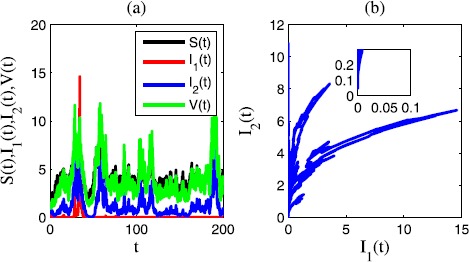
**Time sequence diagram and phase diagram of model** (**4**) **for extinctions of disease 1 and persistence of disease 2.**

In Figure 4, we choose $\sigma_{1}=0.6$, $\sigma_{2}=0.2$, $\sigma_{3}=0.1$, $\sigma _{4}=0.1$, $\gamma_{1}=0.3$, $\gamma_{2}=0.3$, $\gamma_{3}=0.1$, $\gamma_{4}=0.2$. In this case, $R_{1}=-0.0576<0$, $R_{2}=0.0974>0$. We see that in the time sequence diagram Figure 4(a) and the corresponding phase diagram Figure 4(b), the epidemic disease $I_{2}(t)$ is persistent in mean and $I_{1}(t)$ is extinct.

**Figure 5:**
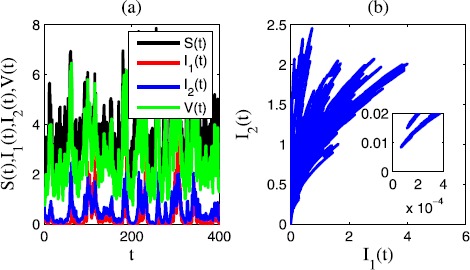
**Time sequence diagram and phase diagram of model** (**4**) **for persistence of two diseases.**

In Figure 5, we choose $\sigma_{1}=0.3$, $\sigma_{2}=0.14$, $\sigma_{3}=0.1$, $\sigma_{4}=0.1$, $\gamma_{1}=0.1$, $\gamma_{2}=0.1$, $\gamma_{3}=0.1$, $\gamma_{4}=0.1$. In this case, $R_{1}=0.0123>0$, $R_{2}=0.0079>0$. We see that in the time sequence diagram Figure 2(a) and the corresponding phase diagram Figure 2(b), the two epidemic diseases are persistent in mean.

Obviously, the numerical simulation results are consistent with the conclusion of our theorems.
